# Utility of contrast-enhanced ultrasound for assessing disease activity in takayasu arteritis with carotid artery involvement: a scoping review

**DOI:** 10.3389/fimmu.2026.1767181

**Published:** 2026-02-05

**Authors:** Carina Soto-Fajardo, Rosa-Elena Cervantes-Ramírez, Carlos-Vidal Montiel-Castañeda, Ada-Rocío Morales-Meza, Daniela-Isabel Rojas-Abarca, Fabiola-Bugambilia Sánchez-Zamudio, Hugo Sandoval, Carlos Pineda

**Affiliations:** 1 Rheumatology Division, Instituto Nacional de Rehabilitación Luis Guillermo Ibarra Ibarra, Calzada México-Xochimilco, Ciudad de México, (CDMX), Mexico; 2 General Directorate, Instituto Nacional de Rehabilitación Luis Guillermo Ibarra Ibarra, Calzada México-Xochimilco, Ciudad de México, (CDMX), Mexico

**Keywords:** carotidarteries, contrast-enhanced ultrasound, inflammation, takayasu arteritis, vasculitis

## Abstract

Takayasu arteritis (TA) is a chronic, immune-mediated vasculitis that primarily affects the large arteries, particularly the aorta and its main branches, leading to stenosis, occlusion, dilation, or aneurysms. Traditional imaging modalities, including computed tomography angiography (CTA), magnetic resonance angiography (MRA), and positron emission tomography/computed tomography (PET-CT), offer valuable diagnostic information; however, their clinical applicability has limitations, including high costs, radiation exposure, and reduced availability. In recent years, contrast-enhanced ultrasound (CEUS) has emerged as a minimally invasive, radiation-free technique capable of detecting vascular wall neovascularization as a potential surrogate imaging marker of TA inflammatory activity.

**Objective:**

This scoping review, conducted in accordance with the Joanna Briggs Institute (JBI) methodology and the Preferred Reporting Items for Systematic Reviews and Meta-Analyses extension for Scoping Reviews (PRISMA-ScR) checklist, aims to synthesize current evidence on the diagnostic and monitoring utility of CEUS in patients with TA and carotid involvement.

**Methods:**

We systematically searched MEDLINE/PubMed, Scopus, Web of Science, LILACS and EBSCO from inception to May 2025 for original studies of adult patients with TA in whom CEUS was used to assess disease activity.

**Results:**

Eighteen studies (8 cross-sectional, 3 cohort, 7 case reports; 631 patients) met the eligibility criteria. Across these studies, CEUS detected arterial wall neovascularization compatible with active inflammation, particularly in the carotid arteries, and demonstrated moderate-to-strong correlations with inflammatory biomarkers, PET-CT findings, and clinical activity scores such as the Indian Takayasu Activity Score (ITAS) and the National Institutes of Health (NIH) criteria. Importantly, CEUS can reveal subclinical or residual inflammation even when clinical symptoms or laboratory markers are absent. Its ability to detect early relapses and monitor therapeutic response has been demonstrated in both observational cohorts and individual case reports. Despite this, substantial heterogeneity in imaging protocols and activity definitions limits comparability across studies and restricts integration into clinical practice.

**Conclusion:**

CEUS is a minimally invasive, reproducible, and effective technique for both detecting and monitoring disease activity in TA with carotid vessel involvement, thereby facilitating early and accurate therapeutic decisions. Its future incorporation into clinical practice will require validated scoring systems, harmonized acquisition protocols, and demonstration of added value in treatment decision-making and long-term outcomes.

## 1 Introduction

Takayasu arteritis (TA) is a rare, chronic inflammatory vasculitis that primarily affects large vessels, particularly the aorta and its major branches, including the ascending aorta, abdominal aorta, renal arteries, and pulmonary arteries. It is more prevalent in women, with a female-to-male ratio of 12:1 in Turkey, 9:1 in Japan, 6.9:1 in Mexico, and 3:1 in China and India. The clinical presentation can be highly variable due to the involvement of each of these arterial segments, making diagnosis a clinical challenge (1–5).

The inflammatory process progresses from the adventitia to the intima, ultimately involving all three arterial wall layers. This results in morpho-structural abnormalities such as stenosis, occlusion, aneurysm formation, or dilation, often leading to downstream ischemia (1, 2, 6).

Numano’s classification system categorizes TA into five variants. Although the prevalence of each variant varies depending on ethnicity and geographical region, involvement of the carotid and subclavian arteries is commonly observed (4, 7, 8).

The clinical management of TA remains challenging, partly because clinical activity scores and conventional biomarkers, such as erythrocyte sedimentation rate (ESR) and C-reactive protein (CRP), correlate poorly with underlying vascular inflammation and disease progression (9, 10).

An accurate assessment of disease activity is still one of the major challenges in the management of TA, as clinical scoring systems (e.g. the National Institutes of Health (NIH) criteria or Indian Takayasu Activity Score (ITAS) 2010 scale) and acute-phase reactants often fail to correlate with true vascular inflammation or angiographic progression (11–13).

Contemporary imaging modalities such as computed tomography angiography (CTA), ultrasound, and magnetic resonance angiography (MRA) provide structural assessment, whereas positron emission tomography/computed tomography (PET-CT) offers metabolic information. However, each technique has limitations. PET-CT, although highly informative, may yield false-negative results in glucocorticoid-treated patients, is costly, and is unsuitable for frequent monitoring (1, 9, 10).

In this context, contrast-enhanced ultrasound (CEUS) has emerged as a promising, radiation-free and cost-effective technique to visualize micro-vascular flow and arterial wall neovascularisation as a surrogate of active inflammation (6, 14–16). CEUS represents a potentially valuable adjunctive tool for evaluating disease activity, particularly in superficially located vessels such as the carotid arteries (3, 6, 16).

However, despite growing interest in CEUS for large-vessel vasculitis, the evidence on its diagnostic performance and role in monitoring disease activity in TA remains fragmented. To our knowledge, no previous review has systematically mapped the available studies, summarized technical protocols and compared CEUS findings with clinical scores, inflammatory biomarkers and established imaging modalities. Therefore, this scoping review aims to synthesize and critically appraise the available evidence on the diagnostic and monitoring utility of CEUS for assessing disease activity in TA.

## 2 Methods

### 2.1 Literature review criteria and search strategy

This scoping review was conducted in accordance with the methodology proposed by the Joanna Briggs Institute (JBL) for these reviews and was reported in accordance with the Preferred Reporting Items for Systematic Reviews and Meta-Analyses extension for Scoping Reviews (PRISMA-ScR) (17, 18). The purpose of employing a scoping methodology was to systematically map existing evidence, summarize heterogeneity in study designs and CEUS techniques, and identify gaps requiring further investigation,

We sought to identify all relevant studies evaluating CEUS for assessing disease activity in TA. A systematic search of original articles was conducted in MEDLINE/PubMed, Scopus, Web of Science, LILACS, and EBSCO, from inception to May 2025. No language restrictions were applied to maximize study capture, following JBL and PRISMA-ScR guidelines (17, 18).

The search strategy was implemented using MEDLINE/PubMed, Scopus, Web of Science, Lilacs, and EBSCO electronic databases. The search strategy combined MeSH terms and free-text terms for “Takayasu arteritis” and “contrast-enhanced ultrasound” and was adapted for each database. In addition, the reference lists of all retrieved articles were manually reviewed. Two independent authors analyzed each article and performed the data extraction independently. In case of disagreement, a third investigator was consulted. Full search strategies are available upon request. The search strategies for the electronic databases are available in the **Supplementary Material**.

### 2.2 Inclusion and exclusion criteria

Eligible studies included observational studies (cross-sectional, cohort, case series) and case reports. Studies involving patients with TA with carotid artery involvement, in which CEUS was used to assess disease activity, were included.

We excluded review articles, animal or *in-vitro* studies, studies limited exclusively to giant cell arteritis, conference abstracts without full-text publication, and studies that did not use CEUS to evaluate disease activity in TA. After removing duplicates, two reviewers independently screened titles and abstracts, followed by full-text assessment of potentially eligible articles. In case of disagreement, a third investigator was consulted.

### 2.3 Risk of bias assessment

We assessed the methodological quality of diagnostic accuracy studies using the Cochrane Handbook for Systematic Literature Reviews and Quality Assessment of Diagnostic Accuracy Studies (QUADAS) 2 tool ​ (19, 20)​. If at least one domain was judged to be at high risk of bias, the study was classified as having an overall high risk of bias; studies with all domains rated as low risk were classified as low risk of bias. Otherwise, if information on the paper was not mentioned, we considered that it has a risk of unclear bias. In the case of case reports and case series, the Joanna Briggs Institute (JBI) Checklists were used​ (21, 22)​. Two reviewers independently performed data extraction and risk assessment of bias, and any disagreements were resolved by a third reviewer.

### 2.4 Data extraction

All the information was collected using a format designed for this review and saved in an Excel sheet. The data collected included: author, year of publication, country, aim, study type, population, age, gender, other demographics, setting, the type and design of the study, number of patients, number of controls, disease compared, comparative diagnostic methods, technical specifications, anatomical regions evaluated and Numano classification.

Regarding technical aspects, the patient’s position, the type of equipment used, and the probe employed were evaluated. In addition, parameters for CEUS acquisition were assessed, such as contrast agent dose, number of boluses, mechanical index, gain setting, focal spot position, image acquisition time, and the scales used for interpretation.

### 2.5 Synthesis of results

Due to substantial heterogeneity in study designs, CEUS acquisition protocols, disease activity definitions, and reference standards, as well as the high level of complexity, a narrative synthesis approach was employed. Quantitative pooling was not feasible and was therefore not attempted; therefore, the results of diagnostic accuracy and correlations are presented only according to the individual studies without pooled diagnostic accuracy.

Owing to the absence of standardized definitions of activity in TA, the reference standard for determining activity relied on clinical judgment, the presence of an NIH score greater than 2 or an ITAS 2010 score >4. Additionally, if an imaging method such as PET-CT was employed, uptake greater than 2 (intermediate, comparable to hepatic levels) was considered indicative of activity. In the case of CEUS, activity was defined by the presence of intima-media neovascularization.

## 3 Results

**Figure 1:**
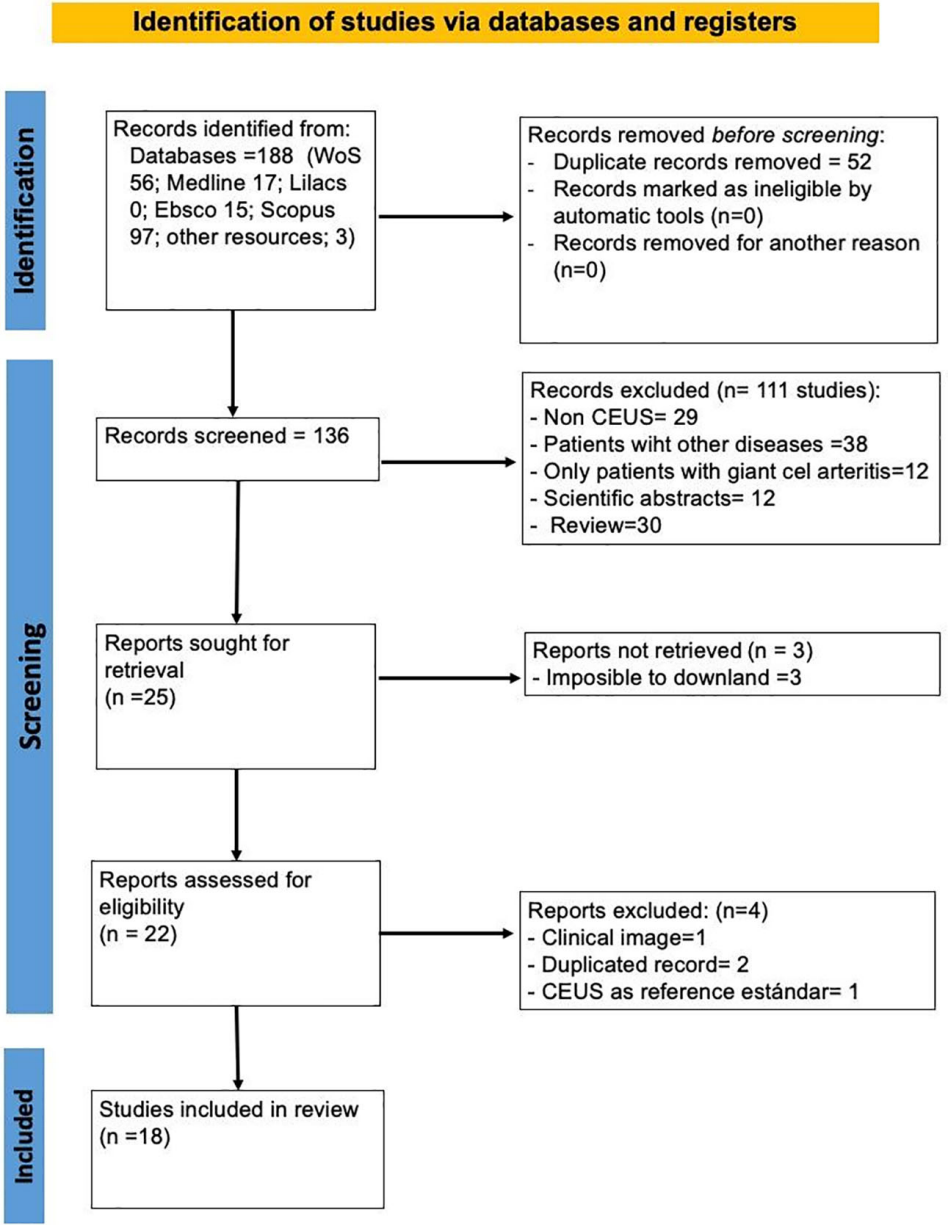
PRISMA flow-diagram.

A total of 109 records were initially identified across the databases. Of the 109 articles identified, after removing duplicates and screening by title, abstract, and full-text review, 18 studies that met the inclusion criteria were included in this revision (**Figure 1**) (23). Of the 18 studies, eight were cross-sectional (313 patients), 5 were case reports (5 patients), 2 case series (8 patients), and 3 were cohort studies that included follow-up (305 patients), representing a total of 631 patients from 7 different countries (China 8, Italy 2, Singapore 1, Germany 2, Switzerland 1, France 3, Netherlands 1), reflecting the limited but heterogeneous evidence base on CEUS in TA. The Numano angiographic classification of TA was reported inconsistently in only 11 of 18 studies. However, patients with types I, II, and V predominated with carotid artery involvement, consistent with published epidemiological patterns. Only two studies (1 cross-sectional and 1 case report) also included patients with LVV.

Follow-up durations ranged from 3 months to 44 months in cohort studies. Regarding case reports, it was shorter and ranged from 3 to 8 months, as reported in 4 of the 7 case reports. **Table 1** summarizes the study type, number of patients, and Numano classification.

**Table 1:** General characteristics of the included studies.

Author	Year	Country	Type	Patients (n)	Numano classification	Follow-up
Germano G (32)	2016	Italy	CS	31 LVV: 14 with TA and 17 with GCA	NR	NR
Hu Y (30)	2017	China	CS	34	NR	NR
Lottspeich C	2018	Germany	CS	40	NR	NR
Huang Y (16)	2018	China	CS	86 (159 CEUS)	I	NR
Li ZQ (6)	2019	China	CS	71	Most type V (62.5%)	NR
Wang Y (34)	2019	China	CS	30	NR	NR
Ding J (15)	2022	China	C	106	I-II= 47 and V = 59	me: 25 (IQR 18-30) m
Goudot G (29)	2023	France	CS	16	I, II, V	NR
Dong Y (3)	2023	China	C	115	I (47), II (28), V (40)	35 patients: 3-6.9m
Zhao C (35)	2018	China	CS	22	NR	NR
Ma L (2)	2019	China	C	84	I (37), II (10), V (37)	38 patients: 3m
Goh Y (31)	2020	Singapore	CR	1	NR	NR
Dikkes A (25)	2017	Switzerland	CR	1	IIB	6d, 3 and 8 m
Herlin B (36)	2015	France	CR	1	I	3m
Giordana P (37)	2011	France	CR	1	I	6m
Magnoni M (27)	2010	Italy	CR	1	V	NR
Czihal M (26)	2017	Germany	CR	3	II	3, 4, and 6 m
Schinkel A (24)	2013	Netherlands	CR	7 LVV: 5 with TA and 2 with GCA	NR	NR

NR, not reported; LVV, large-vessel vasculitis; TA, Takayasu arteritis; CR, Case report or case series; C, Cohort; CS, Cross-section; me= median; IQR, interquartile range; m, month; GCA, Giant cell arteritis.

### 3.1 Assessment of study quality

**Figure 2:**
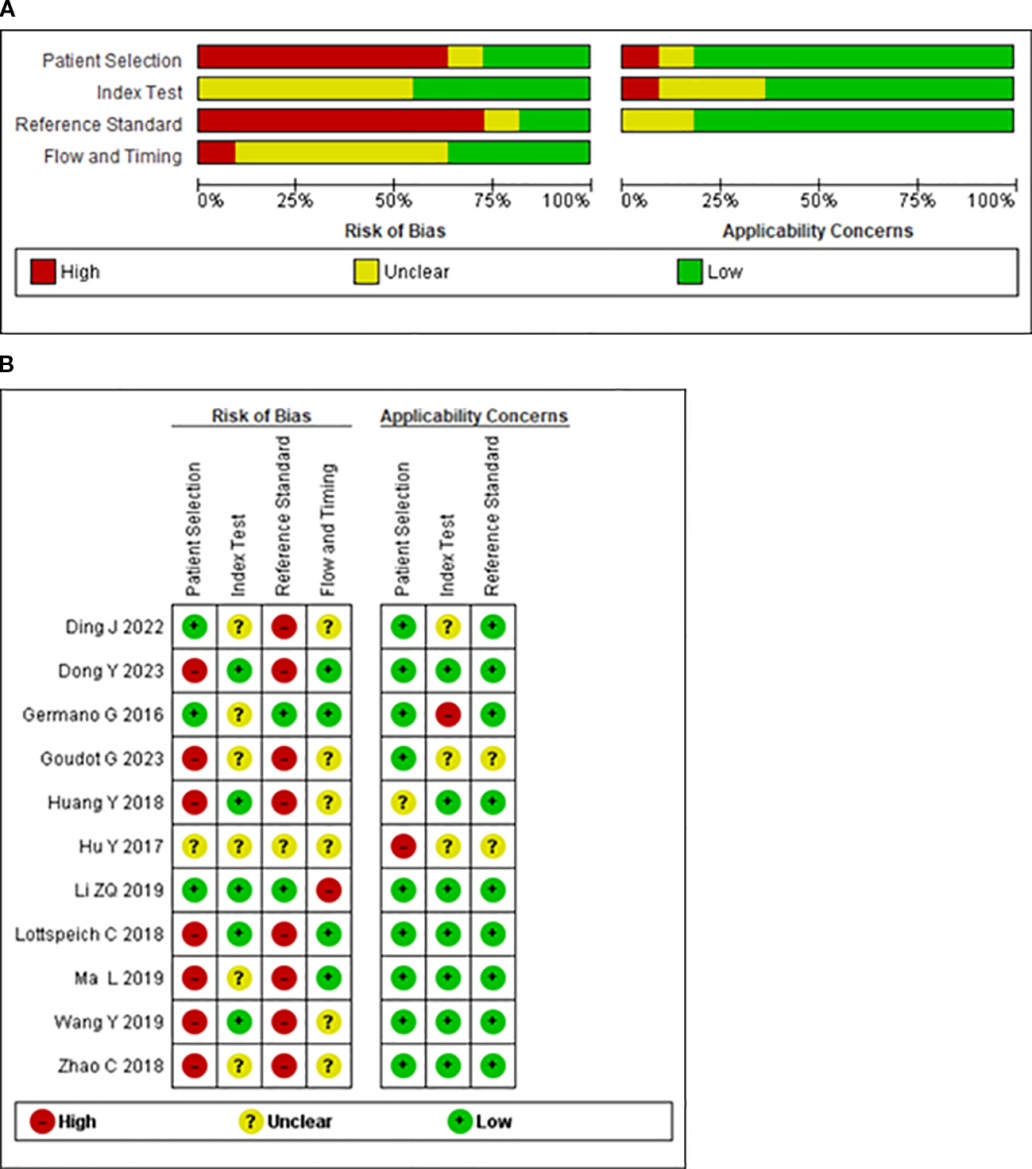
**(A)** Risk of bias and applicability concerns graph: review authors’ judgements about each domain presented as percentages across included studies. **(B)** Risk of bias and applicability concerns summary: review authors’ judgements about each domain for each included study.

Decisions regarding risk of bias and applicability concerns for cross-sectional studies were analyzed using the QUADAS-2 tool, as shown in **Figures 2A, B**. The analysis demonstrates that only two studies presented a low risk of bias in the reference standard domain. This is because most studies used clinical scales as the reference standard. Seven of the evaluated studies had a high risk of bias in patient selection domain because a case-control design was not avoided. Overall, all studies showed a high risk of bias in at least one of the evaluated domains. The results for the quality of case reports and case series are shown in **Supplementary Tables 1** and **2**.

### 3.2 CEUS technical specifications

Only two studies described the patient’s position, which in both cases was supine, with the head at a 45° angle and turned laterally toward the contralateral side being examined, exposing the neck (2, 24).

Among the areas evaluated across the studies, 100% were consistently identified as carotid arteries, evaluated unilaterally or bilaterally. However, some were more specific and explained the territories assessed, such as the common carotid artery, the internal and external carotid arteries, and some even extended their study to the axillary arteries (25, 26).

Regarding the equipment used, there were slight variations among the equipment and brands mentioned, and in the vast majority, linear probes with a frequency range of 3–15 MHz were used.

In general, there was substantial variability among the authors in the CEUS acquisition parameters they used. However, all researchers used a low mechanical index (0.1–0.7), which was not specified in four studies. Only one author specified the software used in their research, known as Contrast Pulse Sequencing. In contrast, most others referred to harmonic contrast software generically or did not mention any specific software. Two different contrast agents were used, Sonovue and Optison (27), with Sonovue being much more frequently used. The volume of contrast agent administered was not reported in six studies, and the remaining studies reported using 0.5-2.5 ml per bolus. This required immediate administration of saline to remove the contrast agent (5–10 ml), as specified in 10 studies. Nevertheless, only three studies reported the number of boluses required. As for the interval between contrast administration and image acquisition, this was specified in eight studies and ranged from 30 to 180 seconds. The images and videos were preserved for later evaluation. These results are summarized in **Table 2**.

**Table 2:** Technical specifications.

Study	Area examined	Equipment	Contrast agent/ Time of infusion	Settings	Number of boluses/Amount	Scan start time	Image interpretation
Germano G 2016 (32)	Common carotid artery, internal and external carotid artery	Esaote MyLab70, 13–5 MHz linear array transducer	SonoVue 5-10s	LMI and contrast software	NR/NR	NR	Semi-quantitative
Hu Y 2017 (30)	Carotid arteries	Philips iU-22 (Philips Medical Systems)	NR	LMI (0,6)	NR	NR	Quantitative
Lottspeich C 2018 (33)	Supra-aortic arteries (1 or 2 segments)	GE LOGIQ E9 (General Electric). 6–15 MHz linear transducer	SonoVue NR	LMI < 0,3. Dynamic range between 0.5 and 0.7.	NR and 2.4ml	NR	Semi-quantitative
Huang Y 2018 (16)	Carotid arteries	Philips iU-22 (Philips Medical Systems) L9–3 MHz linear probe	SonoVue NR	LMI of 0.06–0.1. Imaging contrast-harmonic software.	NR 1.5-2ml + 5ml of saline solution	30s-1m	Semi-quantitative
Li ZQ 2019 (6)	Carotid arteries	Philips iU-22 (Philips Medical Systems) Linear probe L9-3	SonoVue NR	Gain, 30%; Image depth 3cm. NR MI	NR	NR	Semi-quantitative
Wang Y 2019 (34)	Carotid arteries	iU22 (Philips Medical Systems), a 12–5 MHz linear probe	SonoVue NR	MI: 0.06 Image depth: 3-4cm	NR/1.2ml + 5mL of saline solution	10-25s	Semi-quantitative
Ding J 2022 (15)	Thickest site of common carotid artery	iU22 (Philips Medical Systems) Transductor lineal L9-3.	NR	NR	NR	NR	Semi-quantitative
Goudot G 2023 (29)	Carotid arteries	SuperSonic Imagine, SL10–2 linear probe.	SonoVue NR	LMI	NR/2.4 + 10ml of saline solution	Microbubbles detected in the common carotid artery	Quantitative
Dong Y 2023 (3)	Carotid arteries	Philips iU-22 (Philips Medical Systems) L9–3 linear	SonoVue NR	LMI of 0.06	NR/1.2ml + 5ml of saline solution	20-90s	Semi-quantitative
Zhao C 2018 (35)	Carotid arteries	CX50 CompactXtreme ultrasound (Philips Healthcare) 9–13 MHz linear probe	SonoVue NR	Depth 3cm LMI = 0.1	3-4/1.2ml + 5ml of saline solution	3min	Quantitative
Ma L 2019 (2)	Extracranial carotid arteries	Philips Elite (Philips Medical Systems), L9–3 linear probe.	SonoVue NR	LMI 0.07 and Gain 70%.	NR/2ml + 5ml of saline solution	Simultaneously for 2 min	Semi-quantitative
Goh Y 2020 (31)	Carotid arteries	Philips iU-22 (Philips Medical Systems)	SonoVue NR	LMI (0.10)	1/2.5ml + 5ml of saline solution	NR	Qualitative
Dikkes A 2017 (25)	Carotid and axillary arteries	NR	NR	NR	NR	NR	Qualitative
Herlin B 2015 (36)	Carotid arteries	Acuson S2000 (Siemens), 9–4 MHz linear transducer.	SonoVue NR	CPS (Contrast Pulse Sequencing) LMI (0.07)	NR/2.5 mL + 10 mL of saline solution	30s	Qualitative
Giordana P 2011 (37)	Carotid arteries	Siemens S2000, sonda lineal de 9 a 13 MHz.	SonoVue NR	NR	NR/1.5 mL + 5 mL of saline solution	1m	Qualitative
Magnoni M 2010 (27)	Common carotid arteries	GE Healthcare. 7MHz probe.	Optison NR	LMI (0.08-0.1)	NR	NR	Qualitative
Czihal M 2017 (26)	Common carotid and subclavian/axillary arteries	LOGIQ E9 (GE Healthcare), 6–15 MHz linear transducer	SonoVue NR	LMI	1/2.4ml + saline solution	NR	Semi-quantitative
Schinkel A 2013 (24)	Carotid arteries	Philips iU-22 (Philips Medical Systems) L9–3 linear	SonoVue NR	LMI 0.06-0.08 Gain 30%, Compression 60, and Imaging depth 3cm.	NR/0.5ml	NR	Semi-quantitative

MI, mechanical index; LMI, low mechanical index; NR, Not reported.

### 3.3 Use of CEUS in the assessment of disease activity

#### 3.3.1 Defining activity of the reference standard

Definitions of TA disease activity varied markedly across studies. PET-CT was used as the reference standard in only two cross-sectional studies and four case reports. In most of these cases, a semi-quantitative 0–3 scale was used to assess activity based on uptake, where 0 = no uptake; 1 = minimal but not negligible uptake (below hepatic levels); 2 = intermediate uptake (like hepatic levels); and 3 = intense uptake (higher than hepatic levels). In both cross-sectional studies, activity was defined as a grade >2.

On the other hand, in most of the remaining studies, the reference standard was clinical assessment, specifically the NIH criteria or the ITAS 2010 scale. The most frequently used parameter was NIH grade >2.

#### 3.3.2 Defining activity of CEUS

CEUS of the arteries shows the presence of blood vessels with wall neovascularization, which is considered a potential marker of disease activity. As reported by Staub, the presence of blood flow “activity” within the intima-media layer, identified by the dynamic movement of echogenic reflectors (microspheres) within the vasa vasorum, makes it possible to categorize the degree of intima-media neovascularization according to the degree of intraplaque neovascularization (14, 28). In vasculitis, this neovascularization has been associated with disease activity.

In 3 of the studies, neovascularization was assessed quantitatively, for example, in the Goudout study, vascular abnormalities were determined based on the number of microbubbles and their trajectory, resulting in the calculation of the perfusion score (29). Hu Y et al, defined abnormalities using a graph, which reflects the grayscale change of the lesion over time. This graph is called a time-intensity curve (TIC), which can reflect the characteristics of contrast medium intensity (30). Goh Y et al. found moderate neovascularization at the level of arterial wall thickening, leading them to suspect underlying active inflammation of the arterial wall (31). Eight of the cross-sectional studies and two of the case reports, used one of the semi-quantitative scales published by Staub, which assess intraplaque neovascularization. Six of these studies, used the original scale, which range from 0 to 2. In this scale 0 indicates no vascularization, meaning no microbubbles in the media-intima layer. Grade 1 indicates limited or moderate vascularization, and grade 2 indicates severe vascularization (2, 16, 24, 26, 32, 33). In the remaining four studies, the modified scale, ranging from 0 to 3, was used, where grade 1 represents only limited vascularization, grade 2 represents moderate vascularization, and grade 3 represents severe vascularization (3, 6, 15, 34). In two of the aforementioned studies, CEUS activity was defined as a grade >2 on the Staub semi-quantitative scale (0–3) (6, 15). However, when the Staub semi-quantitative scale (0–2) was used, one study defined activity as a grade >1 (33), and another as a grade 2 (32). The remaining studies did not precisely define CEUS activity. Finally, the five remaining case reports did not use any scale to define activity; instead, it was assessed solely qualitatively based on the presence of neovascularization at the evaluated sites. These results are summarized in **Table 3**.

**Table 3:** Evaluation of activity.

Study	Reference standard		CEUS	
	Reference standard	Activity definition†	CEUS scale	Activity definition‡
Germano G 2016 (32)	PET-CT Scale 0-3	Score ≥ 2	Staub 0-2	Grade 2
Hu Y 2017 (30)	NR	NR	Intensity curve (GS) and time (s)	NR
Lottspeich C 2018 (33)	Clinical: NIH and ITAS 2010	NIH >2 plus treatment escalation	Staub 0-2	Grade >1
Huang Y 2018 (16)	NIH	NIH >2	Staub 0-2	NR
Li ZQ 2019 (6)	ITAS 2010 and PET-CT	ITAS 2010 ≥5 or PET-CT >2	Staub 0-3	Grade >2
Wang Y 2019 (34)	NIH	NIH >2	Staub 0-3	NR
Ding J 2022 (15)	NIH	NIH >2	Staub 0-3	Grade >2 and change in image activity
Goudot G 2023 (29)	NIH	NIH >2	Microbubble density and Perfusion score (mm^3^/s^3^)	NR
Dong Y 2023 (3)	NIH	NIH >2	Staub 0-3	NR
Zhao C 2018 (35)	NIH	NIH >2	Enhancement features and time-intensity curves in the region of interest	NR
Ma L 2019 (2)	Clinical	NR	Staub 0-2	NR Follow-up: Decreased vascularization
Goh Y 2020 (31)	PET-CT	NR	NR	NR
Dikkes A 2017 (25)	PET-CT	NR	NR	NR
Herlin B 2015 (36)	PET-CT	NR	NR	NR Follow-up: Decreased vascularization
Giordana P 2011 (37)	PET-CT	NR	NR	NR Follow-up: Decreased vascularization
Magnoni M 2010 (27)	CTA	NR	NR	NR
Czihal M 2017 (26)	MRA	New or progressive concentric wall thickening with gadolinium enhancement	Staub 0-2	NR Follow-up: Decreased vascularization
Schinkel A 2013 (24)	NR	NR	Staub 0-2	NR

PET-CT, positron emission tomography/computed tomography; NIH, National Institutes of Health; ITAS, Indian Takayasu Activity Score; CTA, tomography angiography; MRA, magnetic resonance angiography; NR, Not reported; NA, Not applicable; GS, grayscale; s, seconds.

In summary, definitions of disease activity varied markedly across studies. Reference standards included the NIH criteria, the ITAS-2010 scale, PET-CT uptake (graded 0–3), and clinician judgment. Similarly, CEUS activity thresholds were inconsistent, employing either qualitative criteria (presence of neovascularization) or semi-quantitative scales such as the Staub 0–2 or 0–3 grading systems.

### 3.4 Correlation with biomarkers and clinical scores

Several studies have demonstrated the ability of CEUS to detect active inflammation in TA, particularly in the carotid arteries. Neovascularization, as evidenced by contrast-enhancement, shows a strong association with disease activity (16). In Dong Y et al. study (3), involving 115 patients, extensive enhancement (Grade 3) on CEUS was observed in 67% of those with active disease, compared to only 9% in inactive cases. Moreover, the intensity of enhancement was significantly greater in the active group, suggesting a clear association between CEUS findings and disease activity as suggested by the study by Wang et al. (34).

Additionally, Most cross-sectional and cohort studies demonstrated significant correlations between CEUS enhancement and inflammatory biomarkers (ESR and CRP). For instance, in Li Z study (6), a moderate correlation (r = 0.39/0.32, p = 0.001/0.006) was observed, while in the study by Zhao C et al. (35), a strong correlation between CEUS and ESR/CRP levels was noted (r = 0.88/0.85, p < 0.05). Notably, the strength of this association appears to increase when limited to patients with active or recent-onset disease. This is evident in a study published by Hu Y (30), which included 34 patients, 13 of whom had a recent onset. They found a slight to moderate correlation with ESR (r = 0.34-0.44) and a moderate one with CRP (r = 0.42-0.56) in all patients. However, in the subgroup of untreated patients, this correlation increased slightly in both ESR (r=0.4-0.58) and CRP (0.54-0.76).

### 3.5 Correlation with PET-CT

In addition to inflammatory biomarkers, CEUS has demonstrated a moderate positive correlation with PET-CT. For example, in the study by Goudot G et al., it was demonstrated that CEUS had a moderate correlation with PET-CT (Spearman’s Rho of 0.68, p = 0.004) (29). Similarly, the study published by Germano G et al., it was found that the degrees of vascularization on CEUS showed a moderate correlation with uptake on PET-CT (r=0.773, p<0.001) (32).

CEUS neovascularization showed moderate-to-strong correlation with PET-CT uptake in studies employing metabolic imaging as a reference standard. These findings suggest that CEUS may detect microvascular changes associated with active inflammation.

### 3.6 Diagnostic performance

CEUS has also demonstrated promising diagnostic performance in assessing disease activity. In the study by Germano G et al., CEUS had a sensitivity of 100% (95% CI 65-100%), specificity of 92% (95% CI 72-99%), positive predictive value of 83% (95% CI 51-97%), and negative predictive value of 100% (95% CI 82-100%) when compared against PET-CT (uptake visual grade >2) (32). Similarly, Li Z et al (6) reported excellent diagnostic accuracy (AUC = 0.968), with 100% sensitivity and 80% specificity. It is noteworthy that in this study, CEUS detected inflammation in 56.4% and 48.9% of patients with normal ESR and CRP values. This is particularly relevant for follow-up, as patients may present with persistent symptoms while laboratory results are inconclusive.

Similar performance was observed when CEUS was evaluated against clinical scoring systems such as the ITAS 2010 scale and NIH criteria. In the study by Lottspeich et al., sensitivity was reported as 100% and specificity as 63%. Moreover, combining CEUS with intima-media thickness (IMT) analysis, performance improved substantially, achieving an AUC of 0.99 and sensitivity and specificity of 92.3% and 100%, respectively (33). Similarly, Huang Y et al., reported that CEUS had a diagnostic performance of 0.86 (95% CI 79.7-92.9) with a sensitivity of 84.3% and a specificity of 79.1%; the addition of acute-phase reactants or IMT did not significantly improve diagnostic yield (16).

Further studies corroborate these findings. Dong et al. reported an AUC of 0.81 (95% CI: 0.73–0.90) for Grade 3 enhancement, with a sensitivity of 67% and a specificity of 95% (3). Wang et al. demonstrated that CEUS had high diagnostic accuracy (AUC 0.872, 95% CI 0.785–0.959, p < 0.01) for detecting active disease (34). Finally, Ma L et al., found that CEUS had an AUC of 0.71 (95% CI 0.59–0.83), improving to 0.85 (95% CI: 0.75–0.94) when incorporating wall thickness and ESR, with sensitivity and specificity of 81.1% and 81.5%, respectively (2).

### 3.7 Follow-up and monitoring utility

Due to its minimally invasive and reproducible nature, CEUS makes it an ideal option for long-term monitoring of patients with TA and for assessing treatment response. Therefore, in 7 studies (3 cohort studies and 4 case reports) conducted follow-up assessments over various time frames, ranging from six days up to 44 months, to evaluate the response to treatment through repeat imaging (2, 3, 15, 25, 26, 36, 37). One study included a semiquantitative scale, which categorizes patients presentation as complete (bilateral visual grades improved from active to inactive status), partial (a decrease of≥ 2 or a change from 3 to 2 for the impairment), non-response (no change or a change of ≤ 1 in the total score, indicating an active state), stable (no change or a change of ≤ 1, indicating an inactive state), and relapse (visual grades increased from the inactive to the active state) (15). However, most authors primarily observed a decrease in arterial wall enhancement alongside clinical improvement and reduction of acute-phase reactants after treatment.

Furthermore, in one of the case reports, treatment response was objectively quantified by assessing the grayscale echogenicity of a defined region of interest. Median grayscale values ​​declined markedly, from 80.58 to 5.05, over six months, indicating a significant resolution of inflammatory activity (37).

Additionally, in Dong et al. study, CEUS follow-up was performed on 35 out of 115 patients, including 19 who were clinically active at baseline and 16 who were inactive. During follow-up, it was observed that 89.5% of the active group exhibited a significant reduction in CEUS enhancement, consistent with disease control. In contrast, in the inactive group, 25% showed an increase in enhancement, consistent with a relapse of the disease (3).

One of the main advantages of CEUS for follow-up is its ability to detect residual inflammation even when clinical and laboratory markers are negative. In one clinical case, CEUS identified active inflammation in a patient with negative inflammation markers, leading to a change in treatment that resulted in subsequent resolution of enhancement on follow-up CEUS (36). These findings were also observed in another study (15), in which 106 patients were monitored using CESU. Of these patients 61.3% having more than three assessments (76 active and 30 inactive by CEUS), although 76 patients achieved clinical remission, however CEUS demonstrated remission in only 52 patients, suggesting the presence of ongoing subclinical inflammation in many cases. These results could suggest that CEUS could be used not only for diagnosis, but also for longitudinal assessment, enabling the early detection of relapse and guiding timely therapeutic adjustments.

## 4 Discussion

This scoping review synthesized the available evidence on the use of CEUS to assess disease activity in patients with TA. The findings of this scoping review highlight the emerging yet heterogeneous evidence surrounding the use of CEUS for assessing disease activity in TA. Overall, CEUS has emerged as a potentially useful adjunctive imaging modality; however, the current body of evidence remains fragmented and methodologically inconsistent. While CEUS demonstrates promise as an adjunctive imaging modality, substantial variability in methodology, activity definitions, and reference standards limits its current clinical applicability.

Across studies, CEUS has consistently demonstrated the ability to detect arterial wall neovascularization, a surrogate marker of active vascular inflammation. Increased enhancement patterns were frequently associated with clinical activity, elevated inflammatory biomarkers, and PET-CT uptake. However, these correlations varied widely, reflecting differences in acquisition protocols, contrast dose, probe frequency, MI settings, and timing of image capture.

Regarding Numano classification, types I, II and V were commonly represented, consistent with previous reports (8). Importantly, CEUS assessments were predominantly restricted to the carotid arteries, which may underestimate disease activity in patients with subclavian, thoracic aortic, mesenteric, or renal involvement (Numano III–IV). This anatomical restriction constitutes a structural limitation of CEUS, preventing evaluation of deep or inaccessible vascular territories.

Reproducibility is further limited by inconsistent reporting of technical parameters, including contrast volume, number of boluses, use of saline flush, and the interval between injection and image acquisition (38, 39). These omissions hinder protocol comparison and impede the development of standardized CEUS guidelines.

This review identified a key challenge: the absence of standardized CEUS criteria for defining disease activity in TA. Studies employed multiple semi-quantitative grading systems, including the Staub scale (0–2 or 0–3), as well as qualitative assessments of neovascularization. While the Staub scale has been shown to be associated with disease activity, it must be emphasized that it was originally validated to evaluate atherosclerotic plaques. This raises concerns regarding its applicability in vasculitis, where the pathophysiology of microvascular changes differs substantially (14).

In daily practice, disease activity is determined by clinical judgment; however, some clinical scales, such as the NIH or the ITAS 2010, remain commonly used indices of clinical activity. Both scales have limited sensitivity and specificity, which makes them challenging to use. For instance, the NIH defines disease activity as the presence of two or more items, including elevated acute-phase reactants such as the ESR. However, this could pose a problem, since although an elevated ESR has been correlated with the presence of vasculitis in histopathology, some studies have shown that up to 44% of patients without clinical activity have an elevated ESR. Furthermore, inflammation has also been reported in biopsies despite normal ESR values. Therefore, despite its widespread use, this scale does not adequately represent disease activity. To address this issue, the ITAS 2010 scale and its modification, ITAS-A, were developed; the latter only includes acute-phase reactants. These two scales appear to assess disease activity more accurately than the NIH scale, primarily because they evaluate clinical findings more effectively. However, there are several limitations, including an absence of imaging studies and low predictive value for angiographic progression (11, 40, 41).

On the other hand, several imaging methods have recently been used to assess disease activity, with PET-CT being the most notable. PET-CT appears to be a more effective method of detecting inflammatory activity and offers adequate diagnostic performance. It has therefore proven useful for the clinical assessment of disease activity, predicting relapses, and monitoring treatment responses (11, 40–42).

Recently developed scales that combine PET-CT and clinical findings have demonstrated improved performance and have been shown to predict vascular progression (43, 44). However, drawbacks include the difficulty in performing the test in clinical practice, cost, radiation exposure, and the possibility of false negatives in patients treated with glucocorticoids.

This lack of standardization in the definition of activity with an appropriate reference standard means that the observed correlations may reflect overlapping constructs and limit criterion validity.

Nevertheless, despite heterogeneity across studies, patients with active carotid artery disease showed higher CEUS activity. For example, Schinkel et al. (24) noted that the highest levels of wall enhancement were associated with active TA, while lower levels of CEUS enhancement were observed in inactive TA. It is important to note that histology was not obtained in this study to validate the carotid ultrasound findings, and the results were based on previous studies in patients with symptomatic carotid stenosis undergoing endarterectomy. These studies demonstrated that the atherosclerotic plaque with greater contrast enhancement showed a significantly higher microvessel density in the corresponding region on histology and greater staining for specific vascular and angiogenic markers (45).

Furthermore, in most studies, it has shown a moderate correlation with acute phase reactants. In some studies such as that of Zhao C et al. a strong correlation was found between CEUS and ESR/CRP levels, a difference attributable to the active/inactive status of the recruited patients (35). This association may have limitations, as CRP and ESR are not specific to this or any other rheumatological disease. However, to date, no studies have reported investigating associations with emerging biomarkers in TA disease, such as pentraxin-3, platelet-to-lymphocyte/neutrophil-to-lymphocyte ratio, IL-6, TNF-alpha, IL-8, IL-23, IL-10, IL-18, endothelial damage markers, matrix metalloproteinases, among others (40).

Despite differences in defining activity, one of the most widely used scales was the Staub semi-quantitative scale (0-3, with a cutoff value >2). It is worth noting that this scale was used in the two studies that evaluated diagnostic performance compared to PET-CT, where CEUS demonstrated good diagnostic performance in both studies (6, 32).

Although diagnostic performance was adequate in the studies that compared CEUS with clinical assessment, it was slightly lower across all studies, and sensitivity and specificity varied widely between them. These discrepancies can be explained by the different reference standards used in these studies (determined solely by the clinician or through the use of scales such as the NIH) (2, 3, 16, 33).

Finally, CEUS appears to demonstrate good diagnostic performance in the few individual studies comparing it with PET-CT or clinical activity scores, particularly when combined with intima-media thickness (29, 32). However, it is important to consider that this was only evaluated in a few studies, all with significant methodological biases, making a quantitative synthesis of the evidence impossible and limiting the impact of these results. Hence, the diagnostic performance data are exploratory, and the CEUS cannot currently be considered a validated stand-alone tool or a replacement for PET-CT. Similarly, the lack of prospective validation, small sample sizes, and single-center designs limit generalizability.

### 4.1 Strengths and limitations

A strength of this review is the comprehensive search strategy across multiple databases without language restrictions, which reduces the likelihood of missing relevant studies. In addition, by adopting a scoping review framework, we were able to map technical aspects and diverse definitions of disease activity used in CEUS studies of TA.

However, major limitations affecting interpretation should be acknowledged. First, the overall quality of the included studies was low to moderate, with all diagnostic studies judged to be at high risk of bias in at least one QUADAS-2 domain which limits the certainty of these results. Additionally, due to the nature of this review, case reports and some retrospective studies with methodological limitations and biases were included. Second, the marked heterogeneity in CEUS protocols, vascular territories assessed, and reference standards precluded meta-analysis and limited our ability to compare diagnostic metrics across studies. Third, publication bias cannot be excluded, as most reports originated from specialized centers with a particular interest in CEUS, and negative or inconclusive results may be under-represented in the literature. The inconsistency in disease activity definitions, both for CEUS and the reference standard, further impairs synthesis. Another limitation is that CEUS evaluation was limited in most studies to patients with carotid involvement, which could underestimate disease activity and limiting the universal applicability of CEUS in this population. Finally, due to the heterogeneity of the studies and high methodological biases, it was not possible to perform a quantitative synthesis of the evidence with a deeper exploration of the diagnostic performance of CEUS.

Despite these limitations, the accumulated evidence suggests that CEUS is a promising adjunctive imaging modality for the assessment of disease activity in TA with carotid involvement, warranting further standardization and evaluation in prospective, multicentre studies.

### 4.2 Implications and future directions

Future research should prioritize multicenter prospective studies with standardized CEUS protocols, validation of CEUS-specific scoring systems for vasculitis, incorporation of quantitative perfusion metrics, and exploration of artificial intelligence for automated assessment. Integration of CEUS into multimodal imaging algorithms alongside Doppler ultrasound, CTA, MRA, and PET-based techniques represents an important next step.

## 5 Conclusion

In summary, this scoping review suggests that CEUS is a promising, real-time imaging modality for assessing disease activity in TA. CEUS can visualize arterial wall neovascularization and appears to correlate with clinical activity scores, inflammatory biomarkers, and established imaging techniques, while offering a radiation-free and potentially reproducible option for serial monitoring. Furthermore, CEUS appears to detect subclinical or residual inflammation in some patients whose clinical scores and biomarkers are inactive. These observations suggest that CEUS may complement conventional ultrasound by providing additional microvascular information. However, it is important to emphasize that the current evidence is based on small, methodologically heterogeneous studies at substantial risk of bias, and robust data on prognostic value and impact on clinical decision-making are lacking. In addition, reported diagnostic performance estimates are likely overstated, and prospective validation studies are needed.

Thus, although promising, currently CEUS cannot be considered a substitute for PET-CT or a stand-alone tool for assessing activity. Larger, prospective, multicenter studies with standardized CEUS protocols are needed to define the optimal role of this technique within the multimodal management of TA.

In summary, CEUS is a minimally invasive, reproducible, and effective technique for both detecting and monitoring disease activity in TA with carotid vessel involvement, thereby facilitating early and accurate therapeutic decisions. Its future incorporation into clinical practice will require validated scoring systems, harmonized acquisition protocols, and demonstration of added value in treatment decision-making and long-term outcomes.
