# Supplementary material for: Utility of contrast-enhanced ultrasound for assessing disease activity in takayasu arteritis with carotid artery involvement: a scoping review
Source: Front Immunol. 2026 Feb 5;17:1767181. doi: 10.3389/fimmu.2026.1767181 (PMC12916648; doi:10.3389/fimmu.2026.1767181)
Supplement: Supplementary file 1 [file DataSheet1.docx]

Supplementary Material

# Search strategy

**1.1 MEDLINE (December 2025)**

#1("Takayasu Arteritis"[Mesh]) OR "Arteritis"[Mesh]

# 2 (((contrast-enhanced ultrasound[Title/Abstract]) OR (contrast-enhanced echography[Title/Abstract])) OR (contrast-enhanced ultrasonography[Title/Abstract])) OR (CEUS[Title/Abstract])

#1 AND #2 Filters: Humans, Adult: 19+ years

**1.2 Web of Science (December 2025)**

#1 Contrast-enhanced ultrasound (Topic) and Ceus (OR – Search within topic) and Contrast-enhanced Ultrasonography (OR – Search within topic) and Contrast-enhanced Sonography (OR – Search within topic)

#2 Takayasu arteritis (Topic) or arteritis (All Fields) and Takayasu Arteritis (OR – Search within topic) and Vasculitis (OR – Search within topic) and Large Vessel Vasculitis (OR – Search within topic) and Large-vessel Vasculitis (OR – Search within topic)

Refined By: NOT Document Types: Book Chapters. Click to remove this refine from your search.NOT Document Types: Review Article. Click to remove this refine from your search.

<https://www.webofscience.com/wos/woscc/summary/3f195196-23bc-4302-9dc8-a419c5a06079-01909e5d71/relevance/1>

**1.3 Scopus (December 2025)**

Takayasu arteritis OR arteritis OR vasculitis OR large-vessel vasculitis AND contrast-enhanced ultrasound OR contrast-enhanced ultrasonography OR contrast-enhanced echography OR CEUS AND ( EXCLUDE ( DOCTYPE , "bk" ) OR EXCLUDE ( DOCTYPE , "ch" ) OR EXCLUDE ( DOCTYPE , "re" ) ) AND ( LIMIT-TO ( EXACTKEYWORD , "Human" ) OR LIMIT-TO ( EXACTKEYWORD , "Adult" ) )

# Supplementary Tables

Supplementary Table 1: Quality assessments of case report studies

| Author, year | Q1 | Q2 | Q3 | Q4 | Q5 | Q6 | Q7 | Q8 | Overall Apraissal |
| --- | --- | --- | --- | --- | --- | --- | --- | --- | --- |
| Goh Y 2020 (28) | Yes | No | No | Yes | Yes | Yes | No | Yes | Include |
| Dikkes A 2017 (22) | No | No | Yes | Yes | Yes | Yes | No | Yes | Include |
| Herlin B 2015 (33) | No | Yes | Yes | Yes | Yes | Yes | No | Yes | Include |
| Giordana P 2011 (34) | No | No | No | Yes | Yes | Yes | No | Yes | Include |
| Magnoni M 2010 (24) | No | Yes | Yes | Yes | Yes | Yes | No | Yes | Include |

Supplementary Table 2: Quality assessments of case series studies

| Author, year | Q1 | Q2 | Q3 | Q4 | Q5 | Q6 | Q7 | Q8 | Q9 | Q10 | Overall apraissal |
| --- | --- | --- | --- | --- | --- | --- | --- | --- | --- | --- | --- |
| Czihal M 2017 (23) | Yes | U | Yes | Yes | U | No | Yes | Yes | No | No | Include |
| Schinkel A 2014 (21) | Yes | Yes | U | U | Yes | No | No | Yes | Yes | Yes | Include |

U= unclear
